# Human Peripheral Blood Eosinophils Express High Levels of the Purinergic Receptor P2X4

**DOI:** 10.3389/fimmu.2019.02074

**Published:** 2019-09-06

**Authors:** Viiu Paalme, Airi Rump, Kati Mädo, Marina Teras, Birgit Truumees, Helen Aitai, Kristel Ratas, Mickael Bourge, Chi-Shiun Chiang, Aram Ghalali, Thierry Tordjmann, Jüri Teras, Pierre Boudinot, Jean M. Kanellopoulos, Sirje Rüütel Boudinot

**Affiliations:** ^1^Immunology Unit, Department of Chemistry and Biotechnology, Tallinn University of Technology, Tallinn, Estonia; ^2^North Estonia Medical Centre Foundation, Tallinn, Estonia; ^3^Institute for Integrative Biology of the Cell (I2BC), CEA, CNRS, Univ. Paris-Sud, Université Paris-Saclay, Gif-sur-Yvette, France; ^4^Department of Biomedical Engineering and Environmental Sciences, and Frontier Research Center on Fundamental and Applied Sciences of Matters, National Tsing-Hua University, Hsinchu, Taiwan; ^5^Institute of Environment Medicine, Karolinska Institutet, Stockholm, Sweden; ^6^INSERM U1174 Université Paris Sud, Orsay, France; ^7^Virologie et Immunologie Moléculaires, INRA, Université Paris Saclay, Jouy en Josas, France; ^8^Department of Biochemistry Biophysics and Structural Biology, I2BC-CNRS, Université Paris-Sud, Orsay, France

**Keywords:** P2X4 purinergic receptor, monoclonal antibody, eosinophils, PBL marker, gender difference

## Abstract

Extracellular nucleotides are important mediators of cell activation and trigger multiple responses via membrane receptors known as purinergic receptors (P2). P2X receptors are ligand-gated ion channels, activated by extracellular ATP. P2X4 is one of the most sensitive purinergic receptors, that is typically expressed by neurons, microglia, and some epithelial and endothelial cells. P2X4 mediates neuropathic pain via brain-derived neurotrophic factor and is also involved in inflammation in response to high ATP release. It is therefore involved in multiple inflammatory pathologies as well as neurodegenerative diseases. We have produced monoclonal antibodies (mAb) directed against this important human P2X4 receptor. Focusing on two mAbs, we showed that they also recognize mouse and rat P2X4. We demonstrated that these mAbs can be used in flow cytometry, immunoprecipitation, and immunohistochemistry, but not in Western blot assays, indicating that they target conformational epitopes. We also characterized the expression of P2X4 receptor on mouse and human peripheral blood lymphocytes (PBL). We showed that P2X4 is expressed at the surface of several leukocyte cell types, with the highest expression level on eosinophils, making them potentially sensitive to adenosine triphosphate (ATP). P2X4 is expressed by leucocytes, in human and mouse, with a significant gender difference, males having higher surface expression levels than females. Our findings reveal that PBL express significant levels of P2X4 receptor, and suggest an important role of this receptor in leukocyte activation by ATP, particularly in P2X4^high^ expressing eosinophils.

## Introduction

The purinergic receptors comprise two different groups of receptors, P2Y and P2X. P2X receptors (1–3) are trimeric ATP-gated ion channels (4) which play a key role in neurotransmission, inflammation, and in a variety of other physiological processes. They are encoded by seven genes (*p2rx1 to p2rx7*), corresponding to seven different protein subunits. When ATP is released in a non-regulated manner, it can initiate inflammation and further induce and amplify cell-mediated immunity through P2X receptors (5). Thus, ATP is considered as a danger-associated signal, released from cells during damage, hypoxia, or another cellular stress (6, 7). Extracellular ATP is quickly degraded into ADP, then AMP and finally adenosine by ectonucleotidases present at the plasma membrane of many cells. In chronically inflamed tissues, both extracellular ATP and adenosine may be present at high concentrations for extended periods (8), which suggests a possible role for purinergic signaling in chronic inflammation.

ATP, in its tetra-anionic form, ATP^4−^, can be recognized by myeloid cells via cell surface P2X7 and P2X4 receptors, which leads to Ca2^+^ and Na^+^ influx. While these two receptors are very similar and located close to each other on human chromosome 12, they differ from each other by their ATP binding affinity (from a micromolar range for P2X4 to a millimolar range for P2X7).

The functions of P2X4 are best known in the nervous system [reviewed in (9)]. In particular, P2X4 upregulation in spinal microglia is critical for pain hypersensitivity (allodynia) after peripheral nerve injury (10–12). P2X4 receptors are also involved in alcohol preference (13–15). P2X4 signaling in the CNS is inhibited by ethanol through complex mechanisms leading to altered behavior and promoting the development of alcohol use disorders (16). A broad-spectrum antiparasitic drug named Ivermectin can inhibit this effect of ethanol on P2X4, probably by antagonizing ethanol binding to the purinergic receptor (17, 18).

Data regarding P2X4 involvement in inflammation and immunity are scarce. A potential role of P2X4 in airways pathologies has been reported (19–21). Purinergic signaling has also been linked to allergic asthma (19). Also, in animal models of allergic asthma, elevated levels of P2X4 have been detected in the inflammatory cells of broncho-alveolar lavages (20, 21). While the implication of P2X7 in inflammation has been extensively studied [reviewed in (19, 22)], the characterization of the expression and function of P2X4 in immune cells has been hindered by the lack of Abs directed against this receptor in mouse and human. We therefore undertook the production of mAbs against the human P2X4.

We have produced four monoclonal antibodies (mAb) that recognize human P2X4 specifically. We focused our studies on two of these mAbs [mAb27 (IgG2b) and mAb29 (IgM)], and found that they cross-react with mouse and rat P2X4. Using these mAbs, we characterized the expression profile of P2X4 by human and mouse PBLs. We show that human eosinophils express the highest level of P2X4, while myeloid cells are also positive but express lower amounts of this receptor. Eosinophils are extremely versatile effector cells that damage tissues or modulate the activity of other cells. Eosinophils in homeostasis represent about 1% of circulating leucocytes however that number can rise to 3–5% in inflammatory conditions. In healthy individuals, beside bone marrow and lungs, eosinophils are found in various tissues: ovary, uterus, thymus, spleen and lower gastrointestinal tract. The principal chemotaxins mediating eosinophil recruitment are eotaxins, which attract eosinophils to the gastrointestinal tract, thymus, and uterus and to other organs in disease conditions (23). It has been shown that during chronic inflammation, eosinophils locate close to nerves (24) where activated eosinophils induce nerve damage, neuropeptide release, or altered nerve growth (25). Contact between eosinophils and nerves is one of the causes of airway hypersensitivity in asthma (26). Eosinophils have been considered to be effector cells that are engaged mainly in allergic reactions and in response to parasites. Recently, the role of eosinophils in immunoregulation and homeostasis in the steady state has become more and more evident. A subset of eosinophils (CD16^high^, 1–5% of all eosinophils) have immune regulatory and protective functions since they interact with several immune cell types including dendritic cells and Th1/Th17 lymphocytes (27). The subsets of regulatory eosinophils express the immunoregulatory protein galectin-10, that functions as a T cell—suppressive molecule (28).

In this work, we characterized mAbs specifically recognizing hP2X4, which cross-react with murine P2X4. Our data show that P2X4 is highly expressed by human eosinophils. The P2X4 expression by PBL shows a surprising difference between sexes, males having higher surface levels than females. Our findings pave the way for future studies of the P2X4 role in leukocyte activation by extracellular ATP in normal or pathological conditions.

## Materials and Methods

### Ethics Statement

Animal handling and maintenance were performed according to the interdisciplinary principles and guidelines for the use of animals in research, testing and education (FELASA) prepared by Ad Hoc Committee on Animal Research (The New York Academy of Sciences, New York, NY, USA). The animal experiments described in this study were authorized by the Ethical and Animal Welfare Committee of Estonia (Tartu University, ERC nr 181T-1). The P2X4 KO mice were generated by Sim et al. (29). Human blood samples used in the current study were obtained from healthy donors in accordance with the principles of the Helsinki Declaration of 1975 and subsequent amendments by the World Medical Assembly. Permission No. 160 was issued to Sirje Rüütel Boudinot on 18.02.2013 by the Ethics Review Committee (ERC) on Human Research of the National Institute for Health Development, Tallinn. The patients provided informed consent for data and biological substance collection for usage in clinical research.

### Cell Culture

THP-1 cells (human acute monocytic leukemia cells) obtained from the American Type Cell Culture Collection were cultured in RPMI 1640 GlutaMAX™ medium (Invitrogen, Carlsbad, CA, USA) supplemented with 10% fetal bovine serum (FBS) (Biowest SAS, Nuaillé, France). ALT (mouse astrocytoma), GL261 (mouse glioma), BV2 (mouse microglial cell), and HEK293 (Human Embryonic Kidney 293 cells obtained from Prof Priit Kogerman, TTU, Estonia) were cultured in Dulbecco's Modified Eagle Medium (DMEM) (Invitrogen, Carlsbad, CA, USA) supplemented with 10% FBS (Biowest SAS, Nuaillé, France).

The human prostate carcinoma cell line DU145 was purchased from ATCC (American Type Culture Collection, Manassas, VA). Cells were grown in Dulbecco's modified Eagle's medium (DMEM), with 10% inactivated FBS, penicillin/streptomycin, 1 mM sodium pyruvate. Immortal human bronchial epithelial cells (16HBE14o–) were incubated as described in Cozens et al. (30). In brief, pre-coated conditions were used. Coating was done with bovine serum albumin (BSA, Sigma Aldrich) (0.01 mg/ml), collagen I from bovine 0.03 mg/ml (Corning 354231), human fibronectin 0.01 mg/ml (Corning 354008). Cells were grown in Minimum Essential Medium Eagle with L-glutamine (Lonza, BE12-611F), the medium was supplemented with 10% inactivated FBS, and penicillin-streptomycin. Cell viability in cultures was routinely checked. 16HBE14o– cells were kindly provided by Professor Dieter C. Gruenert, University of California San Francisco (UCSF).

### Stable Transfection of HEK293 Cells

The human or mouse P2X4 receptor-mCherry plasmids (EX-A1754-M56 and EX-Mm24590-M56) were purchased from GeneCopeia and used to tranfect HEK293 cells.

HEK293 cells were transfected using Lipofectamine LTX and Plus^TM^ reagent (Invitrogen) according to the manufacturer's instructions. Stable cell lines were selected in G418 medium and were cultured in DMEM supplemented with 10% FBS and containing 1 mg/ml of G418. Several cell lines expressing the human P2X4 receptor-mCherry or the mouse P2X4 receptor-mCherry were analyzed by flow cytometry. Strongly positive cell lines were frozen down and used for immunoprecipitation experiments.

### Development of Anti-human P2X4 Receptor Monoclonal Antibodies

Mouse mAbs were generated by standard hybridoma technology. C57Bl/6 mice (2 mice, 15 week old females) were immunized intraperitoneally with 40 μg of purified P2X4 HIS-Ectodomain (ECD W50-I339, 289aa not including the HIS-tag) in PBS on days 0, 21, and 42, and boosted without adjuvant on day 59. Spleen and bone marrow cells were then isolated and fused with Sp2/0 myeloma cells 4 days later. Hybridomas producing mAbs against hP2X4 ECD were identified by ELISA, and cloned by the limiting dilution method (31). A detailed description of the protocol is provided in Supplemental Methods. After a second screening based on ELISA and FACS analysis, hybridomas were cultured in 10% FBS DMEM medium. The isotype of mAbs was determined with the Mouse Immunoglobulin Isotyping ELISA Kit (BD Biosciences). IgGs were purified from the supernatant of clones 19 (IgG2b) and 27 (IgG2b) using Pierce Protein G Agarose (Thermofisher Scientific), and conjugated with fluorescein isothiocyanate (FITC, Thermo Fisher Scientific).

### Confocal Microscopy

For visualization of P2X4 on cell lines, cells were fixed with 4% paraformaldehyde Immunostaining was performed using rabbit polyclonal P2X4 antibody [P2X4 (H-40) sc-28764, from Santa Cruz Biotechnology INC. Santa Cruz, CA], mouse polyclonal P2X4 antibody [P2RX4 MaxPab mouse polyclonal antibody (B01) from Abnova Taipei, Taiwan], and our mouse anti-human P2X4 mAb27 (IgG2a/κ). Staining with primary antibodies was performed for 1 h at room temperature. Anti-rabbit or anti-mouse IgG secondary antibodies conjugated with Alexa-Fluor 488 (Invitrogen at Eugene, Oregon, USA) were used. Samples were mounted in DAPI. Fixed cells were analyzed with a Zeiss LSM 510 META confocal laser scanning microscope (Zeiss, Oberkochen, Germany) equipped with a ×63 Plan-oil-immersion lens. An argon laser was excited at 488 nm and fluorescence image was recorded from 500 to 550 nm.

For imaging P2X4 expression by eosinophils in gall bladder from a patient with acute cholecystitis, the tissue was dissected after surgery, cryo-embedded, snap frozen, and stored at −25°C until preparation of cryosections (5 μm). Sections were air-dried, washed with TBS 3 times, and incubated in TBS + 0.2% BSA for blocking. Sections were stained with anti-hP2X4 mAb27-FITC (1:1,600; IgG2b), or mouse anti-h-Siglec-8-PE mAb (1:10; IgG1; from BioLegend clone 7C9). Nuclei were stained using Hoechst33342 (2 μg/ml, Sigma). Control staining was performed using a mouse anti-h CD3-FITC mAb (1:10; clone HIT3a RUO, Isotype IgG2a/, BD Pharmingen). Sections were incubated with Abs in TBS at room temperature for 30 min, washed twice, incubated with Hoechst for 10 min, then washed three times in TBS. Images were acquired on a Zeiss Axioskop2 (10× or a 20× objective) for **Figure 9A**, or a Zeiss LSM780 inverted confocal microscope at 63× for **Figures 9B,C**. Image analysis was performed using ImageJ (NIH).

### Blood Sampling and Leukocyte Isolation

Human samples of venous blood (10 ml) were collected from allergic individuals or healthy controls by venipuncture in the arm into a sterile BD Vacutainer EDTA blood collection tube (BD#367844), at the West Tallinn Central Hospital or at the East Tallinn Central Hospital. Blood samples were kept at room temperature until staining. Regarding mouse PBL, blood samples were collected immediately after death; heparin (100 IU, LEO Pharma, Malmo, Sweden) was used as anticoagulant and cells were stained as described below.

### Flow Cytometry Analysis

#### Extracellular Staining of Cell Lines

Cells were stained with FITC (fluorescein isothiocyanate) or PE (phycoerythrin) conjugated antibodies for 1 h on ice. One million cells were labeled in 100 μl of antibody solution. The following anti-human P2X4 antibodies were used: anti-hP2X4-mAb19-FITC, anti-hP2X4-mAb27-FITC, anti-hP2X4-mAb29 (IgM/κ, hybridoma supernatant or ascites), anti-hP2X4-mAb8 (IgM/κ, hybridoma supernatant or ascites). Secondary Abs were: FITC labeled Goat Anti-Mouse Ig (RUO, GMP, BD Biosciences, cat 349031); Alexa Fluor 488 labeled Goat anti-Mouse IgG, IgM(H+L) (Jackson ImmunoResearch); FITC labeled rat anti-mouse Igκ (clone 187.1 RUO, BD Biosciences, cat 550003). FITC labeled rat IgG2a/κ isotype control (eBR2a) was from eBioscience (cat 11-4321-42). Anti-mouse Fc receptor CD16/CD32 (eBioscience) was used to block Fc-receptors. Data were acquired using a CytoFLEX S (Beckman Coulter) (**Figure 2** and Figure S6) or a BD FACS Canto II (**Figure 3**).

#### Intracellular Staining of Cell Lines

Cells were resuspended in 1 ml of ice-cold Fixation-Permeabilization buffer (eBioscience) and were incubated 60 min in ice and in the dark. Thereafter, 2 ml permeabilization buffer were added and cells were pelleted at (400 x g) 10 min. The cell pellet was re-suspended in 90 μl of ice-cold permeabilization buffer. Reactive groups on cells were blocked with cold DMEM 4% FCS for 20 min. Mouse cells were first incubated with Fc block for 20 min, then stained with the relevant antibodies plus Fc block. Data were acquired using a BD FACS Canto II CS (**Figure 5**).

#### Extracellular Staining of Human PBL

Fluorochrome-labeled antibody mix was added to 50 μl of whole blood (about 2 × 10^6^ leukocytes) and incubated 30 min at room temperature in the dark [for example, anti-P2X4-FITC mAb27, anti-Siglec-8-PE (Nordic BioSite), anti-CD3-PerCP/Cy5.5 SK7 (BD), anti-CD20 PE L27 (BD), anti-CD14-APC cloneMφP9 (BD), anti-CD45-APC-Cy7 2D1 (BD)]. After two washes in PBS, stained samples were treated with FACS lysing solution for 10 min in the dark (Becton Dickinson), which lyses erythrocytes under gentle hypotonic conditions while preserving the leucocytes. Cells were then analyzed by FACS after a final wash in PBS. Eosinophils were identified as: CD45+ Siglec-8+ SSC high. Flow cytometry was performed with BD FACS Canto II (**Figures 6, 7**) and FACS data were analyzed using FlowJo, LLC.

### Statistical Analysis

Tests were performed in Graphpad Prism 8. Experiments were performed at least with six individuals. Results were considered to be statistically significant for *p* ≤ 0.05. A Shapiro–Wilkinson test indicated that data had normal distribution, so Welch's *t-*test was applied to test the significance of differences between groups. A Mann–Whitney test also supported a significant difference between male and female mice groups.

Detailed protocols for hybridoma generation, ELISA, Immuno-precipitation, and western blotting, inhibition of mAb27 binding to the P2X4 receptor by unlabeled anti-hP2X4 mAbs, RNA preparation and RT-QPCRas well as staining of tissue sections are provided in Supplemental Methods.

## Results

### Production of Four Monoclonal Antibodies Against Human P2X4

To generate mouse monoclonal antibodies against human P2X4, a protein comprising the extracellular part of this receptor fused to a 6HisTag was produced in bacteria. It was purified, refolded by dialysis, and used for mouse immunization. Four candidate hybridomas were finally selected, two IgG2b/kappa named mAb19 and mAb27 and two IgM/kappa named mAb29 and mAb8. To test the specificity of the mAbs, Western blot (WB) and immunoprecipitation (IP) analyses from HEK cells expressing the full-length human P2X4 were performed.

**Figure 1:**
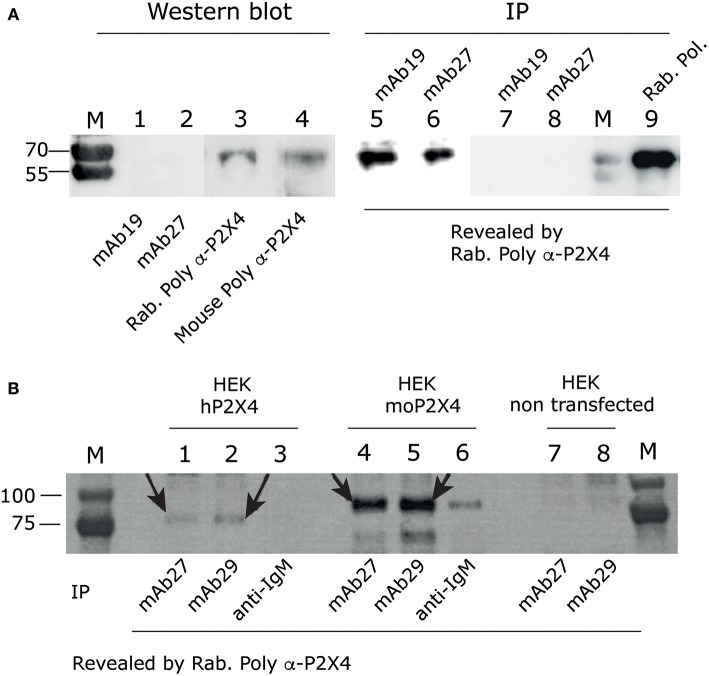
Anti-hP2X4 recognizes hP2X4 in immunoprecipitation experiments, but not in western blot. **(A)** Left panel. Western blot using mAb19 (1), mAb27 (2), rabbit anti-rat-P2X4 polyclonal antibodies from Alomone (3) or our mouse anti-hP2X4 polyclonal antibodies (4), performed on total cell lysate of HEK293 cells transfected with hP2X4. Blots were revealed using anti-mouse and anti-rabbit HRP Abs. Right panel. hP2X4 was immuno-precipitated from the same lysate of HEK293 cells expressing human P2X4, using mAb19 (5) or mAb27 (6), and revealed by western blotting with rabbit anti-P2X4 polyclonal antibodies. Negative controls are shown in lanes 7 and 8: immunoprecipitation of non transfected HEK293 cell lysates with mAb19 (lane 7) and mAb27 (lane 8). Immunoprecipitation of P2X4 from cell lysate of HEK293 cells transfected with hP2X4 using rabbit anti-rat P2X4 polyclonal antibodies is shown in lane 9. **(B)** Immunoprecipitation assays using mAb27 (IgG2b; lanes 1, 4, 7) or mAb29 (IgM; lanes 2, 5, 8) or anti-IgM (lanes 3 and 6) from HEK cells overexpressing hP2X4-mcherry (lanes 1–3), mouse P2X4-mcherry (lanes 4–6), or non transfected (lanes 7, 8). P2X4 was revealed by western blotting with rabbit anti-P2X4 polyclonal antibodies. Arrows indicate hP2X4 or moP2X4 bands.

None of our four monoclonal antibodies detected P2X4 in Western blot experiments (as illustrated in Figure 1A for mAb19 and mAb27, left panel), indicating that they do not recognize the denaturated form of the protein and therefore target conformational epitopes. Similar results were obtained with mAb8 and mAb29 (IgM) (data not shown). In contrast, as shown in Figure 1A (right panel), mAb19 and mAb27 immunoprecipitated P2X4 from the lysates of transfected cells expressing human P2X4, as revealed by staining WB with the commercial rabbit anti-P2X4 extracellular part antibodies from Alomone (polyclonal, cat APR-024). We also asked whether our mAbs could immunoprecipitate both human and mouse P2X4 from lysates of HEK cells overexpressing these proteins (Figure 1B). We then used mAb27 (IgG2b) and mAb29 (IgM) for these experiments and showed that indeed these mAbs could immunoprecipitate both mouse and human P2X4. As observed in Figure 1B, the human and mouse P2X4-mCherry proteins were found at an apparent MW of 81,800 and 89,500 Da, respectively. Thus, our data show that mAb27, mAb19, and mAb29 specifically recognize hP2X4 and that mAbs27 and 29 also bind the mouse receptor. We then mainly focused our studies on mAb27 (IgG2b) and mAb29 (IgM). Hereafter, we show experiments performed with mAb27, indicating when other mAbs were used in parallel.

**Figure 2:**
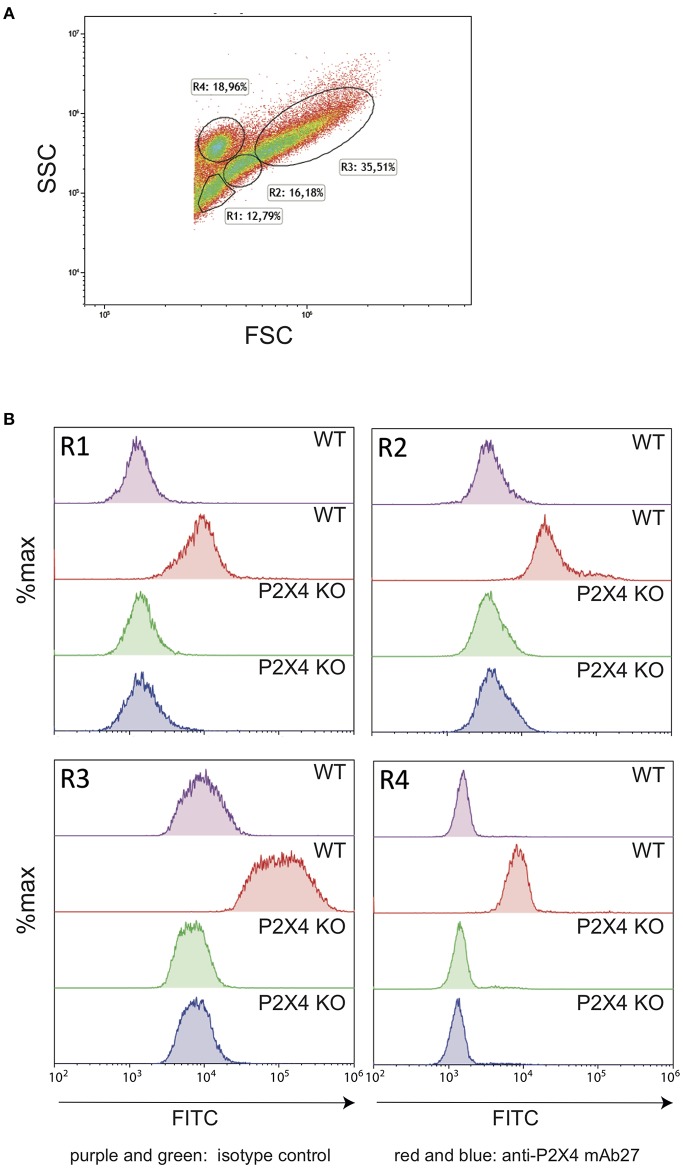
Anti-hP2X4 mAb27 recognizes the mouse P2X4 specifically. **(A)** FSC/SSC representation of mouse peritoneal cells (C57BL/6 strain). Gates were defined as in Hermida et al. (32). Erythrocytes were gated out. During the preparation of cells, erythrocytes were not lysed to avoid ATP release, which would trigger P2X4. Each cell suspension corresponds to a pool from three different mice of each genetic background. **(B)** Comparison of wild type (WT) and P2X4 KO peritoneal cells from gates R1–R4 labeled with isotype control (purple, WT and green, P2X4 KO) or with mAb27 FITC (red, WT and blue, P2X4 KO).

To further confirm that these mAbs recognize the mouse P2X4 receptor, we injected thioglycolate i.p. in P2X4 KO and wild type C57Bl/6 mice. Peritoneal cells were collected and labeled with mAb27-FITC or with isotype control coupled to FITC. Flow cytometry analyses were performed on four main gates (Figure 2A). As shown in Figure 2B, in wild type mice a significant fraction of cells from all gates (R1–R4) were specifically recognized by mAb27. In contrast, in P2X4 KO mice mAb27 staining was not different from the isotype controls. Thus, these results demonstrate that mAb27 can be used to specifically stain murine P2X4 receptor in flow cytometry experiments. Consistent results were obtained with mAb29 (data not shown).

**Figure 3:**
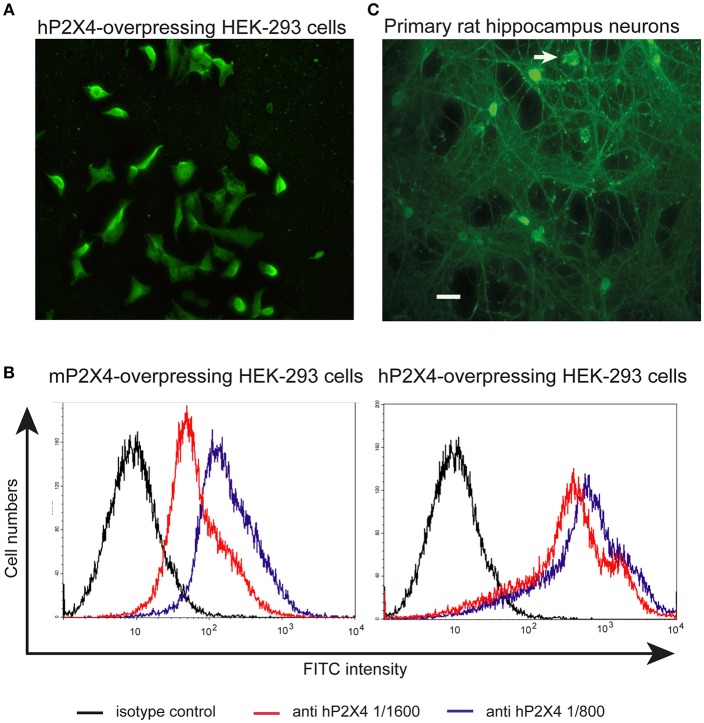
Anti-hP2X4 mAb27 recognizes mouse and rat P2X4 in immunohistochemistry assays. **(A)** Staining of HEK cells transfected with hP2X4 using mAb27 (IgG2b/κ). Scale bar: 15 μm. Staining of HEK cells expressing hP2X4 with secondary Ab alone did not show positive signal (Figure S1). **(B)** HEK cells expressing mouse (left panel) or human (right panel) P2X4 were stained by anti-hP2X4 mAb27-FITC (IgG 2b/κ) diluted 1:1,600 (red line) or 1:800 (blue line). The black line corresponds to staining with the isotype control. **(C)** Primary culture of rat hippocampus neurons with one amoebaean-like glial cell (arrow) was stained with anti-hP2X4 mAb27 (IgG2b/κ). Scale bar: 30 μm. Staining of rat hippocampus neurons with secondary antibodies alone was negative (Figure S1).

The anti-hP2X4 mAbs were further used for immunocytochemistry experiments. mAb27 stained HEK cells overexpressing the human P2X4, but not HEK cells transfected with the empty vector (Figure 3A and Figure S1). mAb19 (IgG2b), mAb29, and 8 (IgM) provided similar results, confirming the specificity of both IgG and IgM anti-hP2X4 mAbs (data not shown). HEK cells were also transfected with an expression plasmid for mouse P2X4; flow cytometry analyses showed that our anti-hP2X4 mAb27-FITC significantly stains cells expressing the mouse P2X4 receptor (Figure 3B, left panel) but less efficiently than cells expressing the human P2X4 receptor (Figure 3B, right panel). Consistent results were obtained with mAb19 (IgG2b), mAb29 (IgM), and mAb8 (IgM) (data not shown). In keeping with this, we used mAb27 to stain rat hippocampus cell cultures containing both P2X4^+^ neurons and glial cells (Figure 3C). Our data are consistent with the high expression of P2X4 in cell bodies of the hippocampus (29). As illustrated for the mAb27 clone (IgG2b) in Figure 3C, these experiments indicate that our anti-human P2X4 mAbs can also recognize the rat P2X4 receptor. Similar images were obtained with the IgM mAb29 (data not shown).

**Figure 4:**
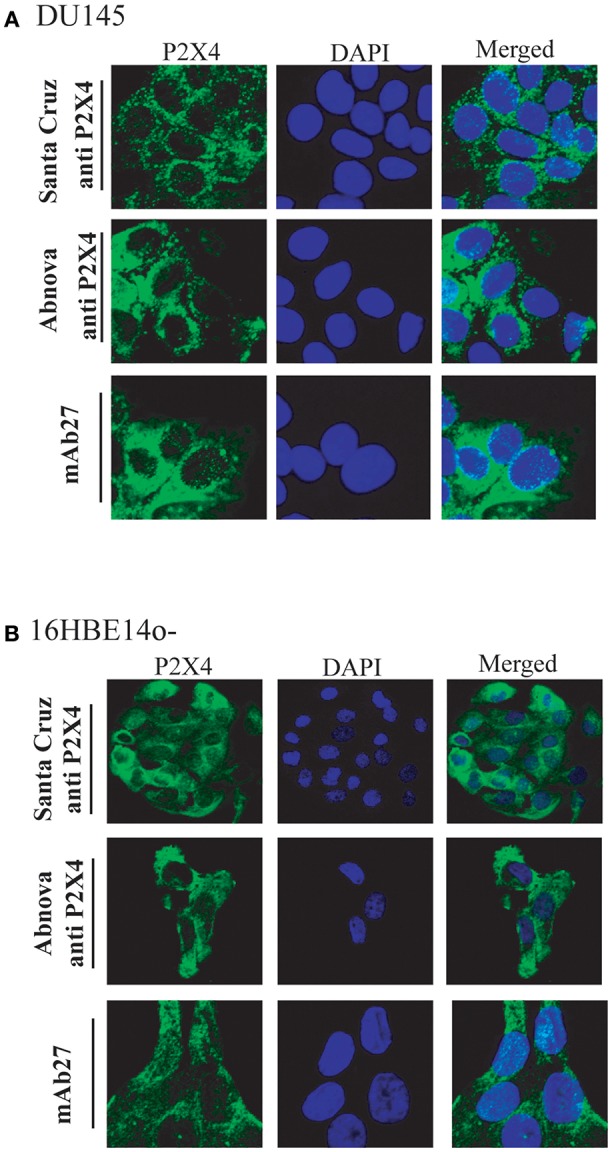
mAb27 staining of human cell lines shows a similar pattern as commercial (polyclonal) anti-hP2X4 Abs. The human prostate cancer cell line DU145 **(A)** or immortal human bronchial epithelial cells (16HBE14o–) **(B)**, were stained with mAb 27 or with two polyclonal anti-P2X4 Abs from Santa Cruz and Abnova. P2X4 localization was visualized by confocal microscopy. Staining with secondary antibodies alone was negative (Figure S2). The images are representative of three independent experiments.

Additionally, a human prostate cancer cell line and human immortal bronchial epithelial cells were stained with the mAb27 and compared with the staining obtained with two other anti-P2X4 antibodies from Abnova and Santa Cruz. As shown in Figure 4 and Figure S2, similar staining patterns were observed, further supporting the specificity of the mAb27.

### P2X4 Is Expressed on the Surface of Mouse Astrocytoma and Glioma Cells but Accumulates Mainly in Intracellular Compartments of Mouse Microglial Cells

We then used our mAbs to study the cellular location of P2X4 in neural cells for which P2X4 expression has been previously demonstrated: astrocytes (33), glia and microglia (34, 35).

**Figure 5:**
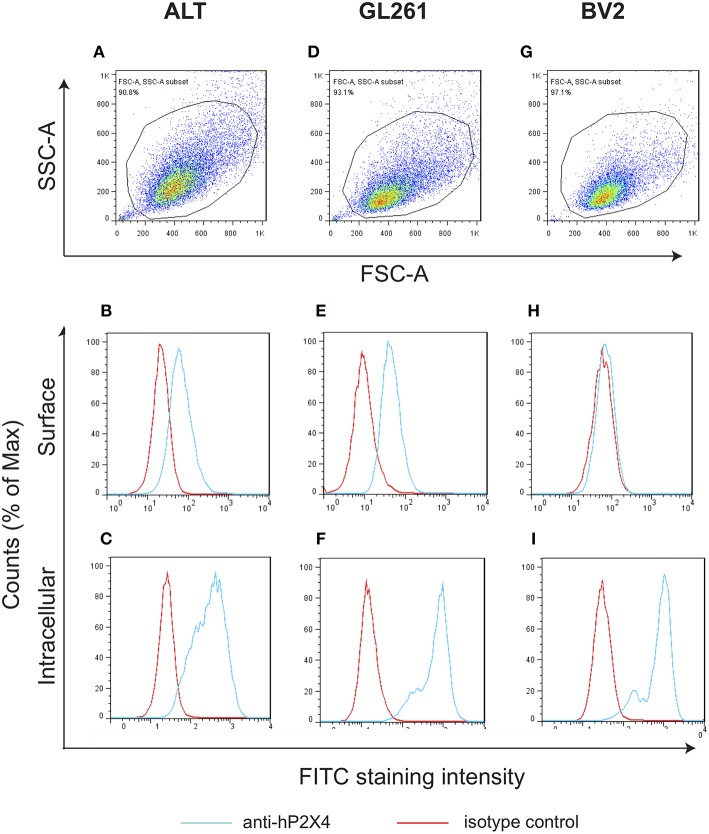
P2X4 expression in several mouse brain tumors is mainly intracellular. Gating strategy of staining of mouse astrocytoma (ALT; **A**), glioma (GL261; **D**); and microglial cell line (BV2; **G**). The percentage of gated cells are indicated. Membrane staining was determined by FACS for mouse astrocytoma (ALT; **B**); glioma (GL261; **E**) and microglial cells (BV2; **H**) with anti-hP2X4 mAb27. Intracellular staining were also analyzed, respectively in mouse astrocytoma (ALT; **C**), glioma (GL261; **F**) and microglial cell line (BV2; **I**). Note that astrocytoma and glioma show surface staining, while microglial cell line has P2X4 receptors only expressed in intracellular compartments **(I)**.

Non-activated mouse brain tumors corresponding to these cell types were stained with purified, FITC-coupled anti-P2X4 mAb27 (IgG2b): an astrocytoma (ALT), a glioma (GL261), and a microglial cell line (BV2). Typical results are shown in Figure 5. Similar results were obtained using mAb19 (data not shown). All cell lines were positive, but the staining revealed a higher surface expression of P2X4 for astrocytoma and glioma, compared to microglial cells which were stained after permeabilization only. BV2 cells showed a strong pattern of P2X4 accumulation in internal compartments, which may correspond to lysosomes as described in COS1 and HEK293 cells (36).

### P2X4 Is Expressed by Different Leukocyte Subsets From Human PBL

**Figure 6:**
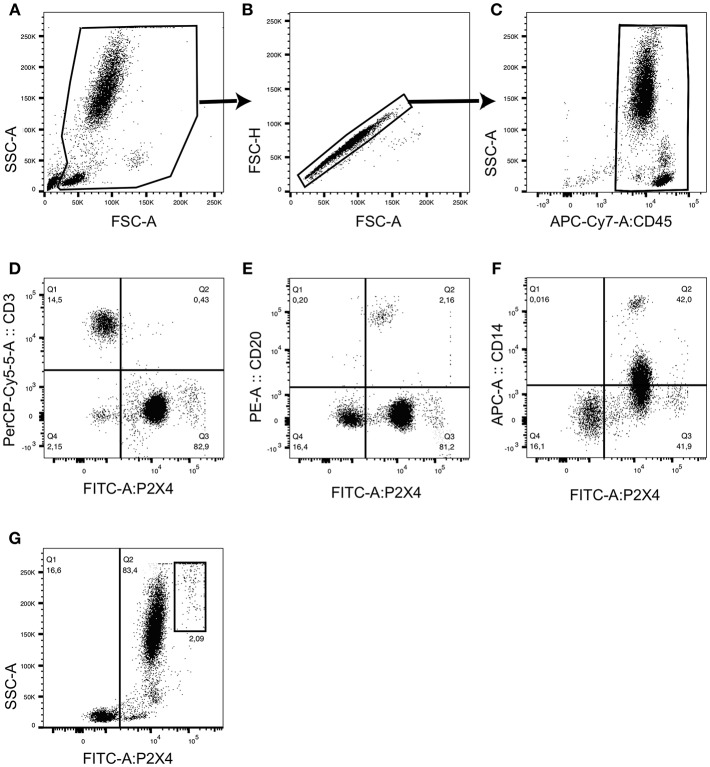
Flow cytometry analyses of hP2X4 expression by CD45^+^ PBL. The gating strategy is presented in **(A–C)**. CD45^+^ leukocytes were stained with fluorochrome-coupled anti-P2X4 mAb27 and with anti-CD3, anti-CD20 and anti-CD14 mAbs. Representative dot plots show P2X4 vs. CD3 **(D)**, P2X4 vs. CD20 **(E)** and P2X4 vs. CD14 **(F)** expressions within the CD45^+^ leukocyte population. A representative dot plot shows the expression of P2X4 vs. side scatter (SSC) and identifies a highly granular P2X4 ^high^ subpopulation **(G)**. This subpopulation represented about 2% of the CD45^+^ leukocyte population. Quadrants were placed based on the fluorescent levels observed in isotype controls. Additional controls are shown on Figure S3.

To investigate a possible role of P2X4 in immune cell activation, we then evaluated the P2X4 expression by leukocytes from peripheral blood of 7 healthy donors, defined as CD45^+^cells. PBL were stained with purified, FITC-coupled anti-P2X4 mAb27, and with lineage-specific antibodies (CD45, CD20, CD3, CD14), and analyzed by flow cytometry to assess the expression of P2X4 by the main leukocyte subsets (Figure 6). Within CD45^+^ cells, flow cytometry data showed that CD3^+^ T cells expressed very low levels of P2X4 (Figure 6D), while CD20^+^ B cells were slightly positive (Figure 6E). This was observed in all individuals studied but one, where P2X4 expression level was similar in B and T cells, at a very low level. In contrast, CD14^+^ monocytes and CD14^+/−^ granulocytes cells expressed intermediate levels of P2X4 (Figures 6F,G). Interestingly, a distinct subset expressing a high level of P2X4 appears in Figures 6D–F. As shown in Figure 6G, these cells were among the most granular cells in PBL, and expressed P2X4 receptor at a much higher level compared to other granulocytes. Overall, these observations identified a cell subset expressing high level of P2X4 that was distinct from T and B cell, monocytes, and the main fraction of granulocytes.

### Human Eosinophils Express High Levels of P2X4

**Figure 7:**
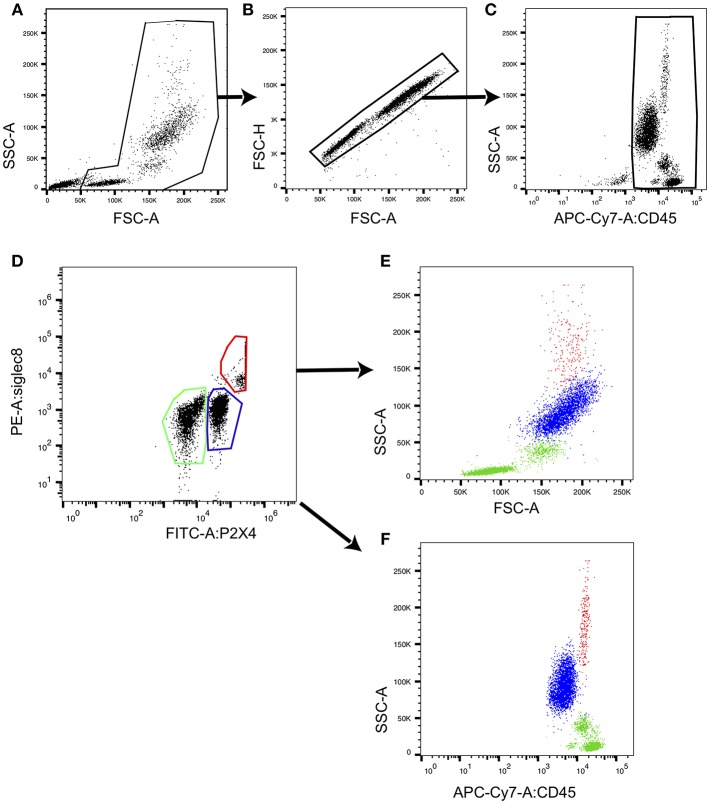
Eosinophils from human PBL express highest levels of P2X4. CD45^+^ peripheral blood leukocytes defined by the gating strategy presented in **(A–C)** were stained with fluorochrome-coupled anti-P2X4 mAb27 and with anti-Siglec8 mAb, and analyzed by flow cytometry. A representative dot plot shows P2X4 vs. SIGLEC8 expression **(D)** in CD45^+^ leukocytes. Three gates outlined in red (gate 1), blue (gate 2), and green (gate 3) define P2X4^high^SIGLEC8^high^, P2X4^med^SIGLEC8^low^, and P2X4^low^SIGLEC8^low^ subsets, respectively. **(E,F)** Represent forward (FSC) (respectively, CD45 expression) vs. side scatter (SSC) for cells from each gate, showing that P2X4^high^SIGLEC8^high^ are large, highly granular cells.

We then characterized a population of large, highly granular P2X4^high^CD45^+^CD3^−^CD14^−^ cells observed in human PBL by flow cytometry analyses. From their phenotype and frequency, we hypothesized that these cells might be eosinophils. Eosinophils are typically abundant among PBL of allergic patients. We therefore performed staining of human PBL from six allergic donors using anti-CD45, anti-P2X4 mAb27, and anti-Siglec8 mAbs, and analyzed these cells by flow cytometry. In Figure 7, representative dot plots show that the subpopulation of highly granular P2X4^high^ cells corresponds to the cell subset expressing high level of the eosinophil marker Siglec-8. In comparison, lymphoid or myeloid cells expressing intermediate or low levels of P2X4 all express lower levels of Siglec-8 (Figure 7D): P2X4^med^Siglec8^low^ cells are granular, large cells with intermediate levels of CD45 and correspond mainly to neutrophils, while P2X4^med/low^Siglec8^low^ cells are smaller and express high levels of CD45, comprising monocytes and lymphocytes (Figures 7D–F). In addition, the P2X4^high^ population of PBL from allergic patients expresses low levels of CD123 and is clearly distinct from the CD123^+^ basophils (Figure S4).

Relative frequencies of P2X4^high^Siglec8^high^, all P2X4^high^ and all Siglec8^high^ cells were measured from PBL of nine patients allergic to pollen (2), mold (2), honey (1), mites in house dust (1), mice (1), and one patient with an acute allergic dermatitis. The results are summarized in Table 1, and indicate that a high surface expression of P2X4 is a good marker for Siglec-8^+^ cells in human PBL. Taken together, our data show that the frequencies of PBL large granular cells with a strong surface expression of P2X4 correlate with the frequencies of Siglec-8^high^ cells in allergic patients.

**Table 1:** Human PBL eosinophils express high P2X4 levels at the cell surface^a^.

**Sex and age**	**Known allergy**	**% of DP Siglec8^**high**^ P2X4^high^^b^**	**Total** **% of P2X4^high^^b^**	**Total %** **of Siglec8^high^^b^**
F, 21	Pollen, cats, mites^*^	1.08	1.08	1.12
F, 50	Honey, propolis^**^	0.9	0.9	1
M, 48	Mold^**^	3.94	3.95	4.2
F, 54	Nickel^*^	1.9	1.95	1.89
F, 51	Pollen, citrus^**^	5.6	5.7	5.7
F, 56	Atopic dermatitis^**^	2.23	2.23	2.25
F, 35	Mice^*^	0.78	0.93	0.96
M, 49	Mites and molds^**^	4.62	4.64	5.1
M, 44	Not specified^*^	1.08	1.7	1.09

* Currently stable;

** *Currently reactive*.

a *Anti P2X4 mAb27 was used for these experiments*.

b *Percentages of P2X4^high^SIGLEC8^high^DP, P2X4^high^, and SIGLEC8^high^ within the CD45^+^ leukocyte population as defined in Figures 6A–C*.

**Figure 8:**
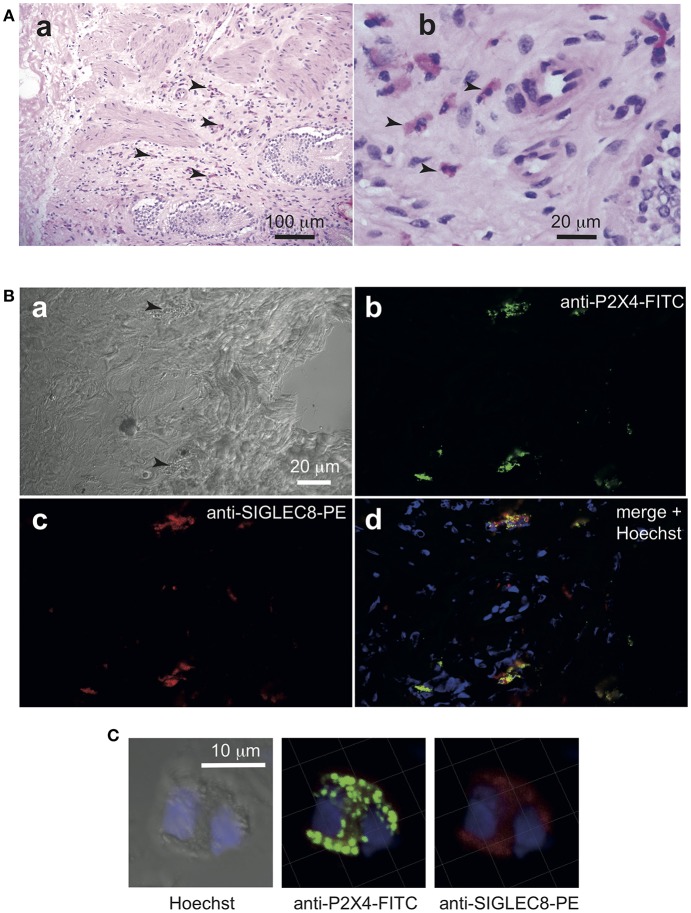
Anti-hP2X4 staining identifies eosinophils in gall bladder sections. Cryosections (5 μm) of freshly isolated gall bladder sample from a patient with cholecystitis diagnosis were stained by hematoxylin-eosin **(A)**. Eosinophils are indicated by arrows. In **(B)**, two granulocytes are indicated by black arrows in the bright field image (a). Sections were stained with anti-hP2X4-FITC mAb27 (b), anti Siglec-8-PE (c). Merged images with Hoechst staining is shown in (d). Images acquired by confocal microscopy indicate that Siglec8-positive eosinophils express high levels of P2X4. **(C)** Illustrates the respective intracellular distributions of Siglec-8 and P2X4. Isotypic control is shown in Figure S5.

Analysis of the gallbladder surgical specimen from a patient with chronic calculous cholecystitis (Figure 8 and Figure S5) shows specific co-staining of P2X4 and Siglec-8, indicating that eosinophils in tissues express those markers at high level. Another section of the same sample was stained with hematoxylin/eosin to visualize the density of eosinophils in the tissue. These results indicate that the anti-hP2X4 mAb27 can be used to visualize eosinophils in inflammatory tissues.

### P2X4 Is Expressed by a Higher Fraction of Leukocytes in Males Compared to Females

P2X4 functions in brain microglial cells have been shown to be sex-dependent. Upregulation of P2X4 expression by these cells is required for pain hypersensitivity in male mice, but not in females where lymphocytes may be implicated (37, 38). We therefore compared P2X4 expression on PBL from men and women, as well as from male and female mice using our mAb27.

**Figure 9:**
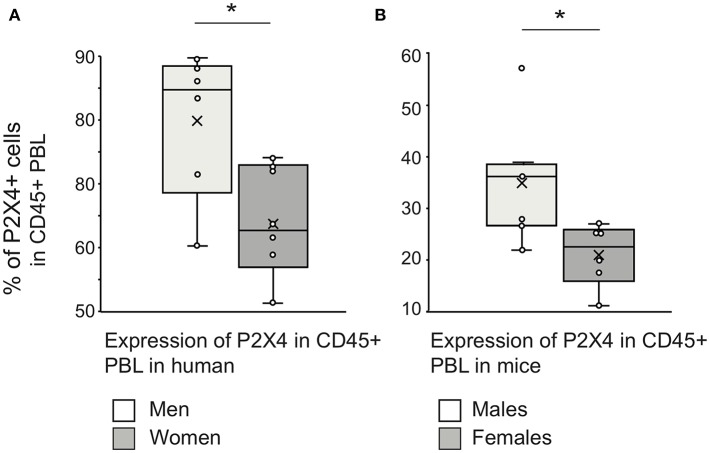
Gender difference in P2X4 expression by peripheral blood leukocytes. Whiskers plots of percentages of CD45^+^P2X4-positive within PBL of healthy men (*n* = 6) and women (*n* = 6) **(A)**, or within PBL of healthy males (*n* = 6) and females (*n* = 6) C57BL6 mice **(B)**. The percentage of P2X4-positive cells was measured using flow cytometry, the cut-off threshold being determined from staining using isotype control. The “X” sign denotes the mean value, the horizontal bar the median, and the whiskers show maximum and minimum values. The “^*^” sign denotes a statistically significant difference (*p* < 0.05) between the compared groups. Welch's *t*-test was applied to test the significance between groups (human: *p* = 0.021; mouse: *p* = 0.022), since a Shapiro–Wilkinson test supported that data had normal distribution. Additionally, a Mann–Whitney test supported a significant difference between male and female mice groups (*p* = 0.014).

Men had a higher fraction of P2X4 positive cells compared to women, when all blood CD45^+^ leucocytes were considered (Figure 9A). Similar experiments were then performed with mouse PBL, to test whether this expression pattern across leukocytes was conserved between human and mouse. The results showed a similar distribution as in humans, with lower P2X4 expression in females (Figure 9B). It is also interesting to note that the biggest percentages of Siglec8^high^P2X4^high^ cells identifying eosinophils were observed in two male patients (Table 1).

Overall, these results show that human and mouse leukocyte subsets express significant levels of P2X4, suggesting that this purinergic receptor can play a role in their activation. The percentage of P2X4 positive cells appears to be consistently higher in men and male mice.

## Discussion

In this work, we report the production and validation of several mAbs against human P2X4. We characterized mAb27(IgG2b) and the mAb29(IgM), and showed that they cross-react against the murine ortholog of this receptor. We used mAb27 to assess the expression of P2X4 on leukocytes. We demonstrated that high expression level of P2X4 is an excellent surface marker for human eosinophils (Siglec-8^high^ cells), in PBL of healthy individuals and allergic patients. We also observed that the expression levels on leukocytes were higher in males compared to females, in mouse and human.

Purinergic P2X receptors are membrane channels that bind extracellular ATP and mediate most of its functions (39). Their roles are partly redundant but they do not have similar expression patterns across tissues and cell types (2, 40). It is therefore important to generate specific reagents such as mAbs to determine their expression range and functional capacity. Antibodies raised against synthetic peptides usually work in Western blot but fail to bind the native protein. In contrast, our mAbs were produced after immunization with the hP2X4 extracellular domain and screened using eukaryotic cells expressing hP2X4. Multiple assays including flow cytometry of transfected cells, immunoprecipitations, and immunochemistry show that these mAbs are specific for hP2X4, and recognize this receptor in native conformation. They did not work in Western blot assay, which is consistent with the presence of disulphide bonds (S-S) and N-linked glycosyl chains in the extracellular domain of P2X4 (41). Intracellular patterns of IHC with our mAbs were very similar to those obtained with commercial polyclonal Abs. The specificity of mAb27 for mouse P2X4 is clearly established by its capacity to label peritoneal cells from WT mice, but not those from P2X4 KO animals. Importantly, we also observed that our anti-hP2X4 mAbs did not cross-react with HEK cells expressing human P2X7 (Figure S6)—the member of P2X family that is the most similar to P2X4—further establishing their specificity to the P2X4 receptor. In addition, we found that preincubation of cells expressing hP2X4-mcherry or hP2X7 with mAb27 or mAb29 inhibited the binding of mAb27-FITC to cells expressing hP2X4-mcherry only. Thus, mAb27 binds specifically to P2X4, and binds the same or a closely located epitope as mAb29. Human and mouse (or rat) P2X4 amino-acid sequences are about 87% similar to each other, thus it was interesting to test whether our mAbs cross-react with murine P2X4 specifically. We indeed showed that the mAbs can recognize the mouse receptor in flow cytometry, immunoprecipitation, and IHC experiments. This highlights the potential use of our mAbs in murine species.

Interestingly, three monoclonal antibodies against the ectodomain of the rat P2X4 receptor have been raised previously by two other groups (42–44). These mAbs reacted with the rat P2X4 receptor in its native conformation and could immunoprecipitate it. However, only the mAb raised by Bo et al. was tested on the human P2X4, and it did not recognize it (42). Thus, our mAbs specific for the human P2X4 and cross-reacting with the murine ortholog represent novel tools allowing studies on the presence and function of this receptor in several tissues and PBL, particularly eosinophils.

Using our anti-P2X4 mAb27, we showed that this purinergic receptor is expressed on the surface of leukocytes (defined as CD45^+^ cells), at variable levels across cell types. We found that the overall P2X4 expression by PBL and spleen leukocytes was significantly higher on males, compared to females. Importantly, this difference stands for both mice and humans, indicating that sexual dimorphism could be a fundamental feature of the conserved, hence essential, functions of the receptor. This was observed consistently in the context of a significant difference in the overall proportion of P2X4 expressing leukocytes between human (60–80%) and mouse (20–40%), reflecting the low frequency of neutrophils in mouse blood. Interestingly, differences in the P2X4 functions between sexes have been documented, regarding neuropathic pain. While the P2X4 dependent microglia-neuron signaling is required for chronic pain hypersensitivity in males (39, 45) microglia do not contribute to pain hypersensitivity in female mice. Allodynia is in fact abolished in Rag 1 KO female mice, indicating that it requires the presence of adaptive immune cells (38). In females, peripheral nerve injury (PNI) induces an upregulation of P2X4 receptor in spinal dorsal horn, but at a much lower level when compared to males (46). It will be interesting to explore further whether a higher expression of P2X4 by myeloid cells from the blood or spleen of males may also participate to pain signaling. Taken together, all these observations raise the question of a possible sexual dimorphism of the functions mediated by P2X4. Interestingly, a recent study showed that deletion of P2X4 receptor was neuroprotective, and enhanced recovery from ischemic stroke in female mice only (47). Whether or not the sexual dimorphism of P2X4 expression on leukocytes is important for human pathologies remains to be clarified.

Flow cytometry experiments using our anti-P2X4 mAb27 revealed that this purinergic receptor is highly expressed by large, granular cells with high levels of the Siglec-8 surface marker. Additionally, we verified by RT-PCR that sorted CD45^+^ P2X4^high^ cells from human PBL expressed Siglec-8 mRNA (Figure S7). This sialic acid-binding lectin is strongly expressed by eosinophils (48), by mast cells and to a lesser extent by basophils (49, 50). In human PBL, the P2X4^high^ cells do not correspond to mast cells that are not present in blood (51–53), which strongly suggests that they are eosinophils. In addition, P2X4^high^ cells in gallbladder sections were clearly eosinophils and not mast cells. Like mast cells, basophils are very rare among human PBL (54). In allergic patients, a basophil population can be detected as CD123^+^ cells, but they express only low levels of P2X4, supporting that P2X4^high^ cells are eosinophils (Figure S4). Other granulocytes (essentially neutrophils), monocytes/macrophages (CD45^+^, CD14^+^) and B cells (CD45^+^, CD20^+^) express intermediate levels of P2X4, while T lymphocytes are negative. These results confirmed previous observations reporting that P2X4 was expressed by myeloid cells, in particular by microglial cells in the brain. We also observed a strong surface expression of P2X4 on eosinophils in PBL of both healthy donors and patients with allergic pathologies. In these individuals, eosinophils are 2–3 times more frequent in PBL than in healthy people (51). Hence, the strong P2X4 expression by eosinophils is also seen on circulating and activated cells during allergy. Since P2X4 detects extracellular ATP at micromolar concentrations, these observations suggest that eosinophils may use this receptor for ATP-induced activation.

Eosinophils are terminally differentiated cytotoxic effector cells that control immune homeostasis and exert immunomodulatory functions. They provide host protection against parasitic, fungal, bacterial, and viral infections (55, 56) but also contribute to tissue damage during infections, asthma and autoimmune diseases. The differentiation of eosinophils from progenitor cells is controlled by inflammatory stimuli such as IL3, IL5, and CCL11/eotaxin; mature eosinophils are released to the blood (26) and infiltrate thymus, spleen and lymph nodes, as well as Peyer's patches and bone marrow where they promote plasma cell survival (57). Our results suggest that extracellular ATP, even at low concentration, may play a role in eosinophil activation, maybe at the degranulation step through which these cells release active compounds.

Although eosinophil count in blood and biopsy samples may not correlate with disease severity, there is a clear evidence of eosinophil activation during multiple pathologies. Eosinophils are implicated in allergic diseases as well as in asthma and in antiparasitic immune responses (58). The P2X4 expression by eosinophils should be considered in conjunction with recent reports showing that this purinergic receptor enhances allergic responses (20, 21). However, the immune functions of these cells remain poorly understood, partly because of the lack of specific surface markers and tumor cell lines. As a new surface marker of human eosinophils, P2X4 appears as a useful target to get insight into their biology. Importantly, Siglec-8 cannot be used as a human cell marker, in *in vitro* culture or *in vivo* studies because its engagement by specific antibodies results in apoptosis of eosinophils and inhibition of mediator release from mast cells (50), highlighting the importance of new markers for this cell type.

## Conclusion

In this work, we have produced and validated anti-P2X4 mAbs. Using the IgG2b mAb27, we show that expression level of P2X4 by myeloid cells is higher in males compared to females, with potentially important consequences for several pathologies. We also report that among human PBL, eosinophils are by far the cell type expressing the highest level of P2X4 on the cell surface, suggesting that ATP-dependent activation could be important in the eosinophil biology in healthy and pathological contexts.

## Ethics Statement

Animal handling and maintenance were performed according to the interdisciplinary principles and guidelines for the use of animals in research, testing and education (FELASA) prepared by Ad Hoc Committee on Animal Research (The New York Academy of Sciences, New York, NY, USA). The animal experiments described in this study were authorized by the Ethical and Animal Welfare Committee of Estonia (Tartu University, ERC nr 181T-1). Blood samples used in the current study were obtained from healthy donors in accordance with the principles of the Helsinki Declaration of 1975 and subsequent amendments by the World Medical Assembly. Permission No. 160 was issued to SB on 18.02.2013 by the Ethics Review Committee (ERC) on Human Research of the National Institute for Health Development, Tallinn.

## Author Contributions

VP, AR, JK, and SR conceived the project. VP, AR, HA, KR, AG, JT, C-SC, MB, JK, and SR designed experiments. VP, AR, KM, HA, KR, BT, AG, C-SC, MB, JK, and SR performed wet-lab experiments. VP, AR, KM, MT, AG, JT, C-SC, PB, MB, JK, and SR performed primary data analysis. HA, KR, MT, BT, AG, JT, AR, JK, TT, and C-SC provided resources. VP, MT, PB, JK, TT, and SR wrote the manuscript. All authors edited the manuscript.

### Conflict of Interest Statement

The authors declare that the research was conducted in the absence of any commercial or financial relationships that could be construed as a potential conflict of interest.
