# Supplementary material for: Human Peripheral Blood Eosinophils Express High Levels of the Purinergic Receptor P2X4
Source: Front Immunol. 2019 Sep 6;10:2074. doi: 10.3389/fimmu.2019.02074 (PMC6746186; doi:10.3389/fimmu.2019.02074)
Supplement: Supplementary file 1 [file Data_Sheet_1.pdf]

Supplemental methods.

Hybridoma generation.

The human P2X4 (hP2X4) full-length cDNA was cloned using RT-PCR: total RNA was extracted from LPS-activated THP-1 cells, reverse transcribed with RevertAid Reverse Transcriptase (Thermo Fisher Scientific) and amplified with Taq DNA Polymerase (Thermo Fisher Scientific) using sequence-specific primers (forward 5′-CACGGATCCACCATGGCGGGCTGCTGCG–3’ and reverse 5′-CACCTCGAGTCACTGGTCCAGCTCACTAGC-3’) based on sequence data from Ensembl (Ensembl ID: CCDS9214.1). The amplified cDNA was cloned into pcDNA3.1 (Invitrogen) in *BamHI*/*XhoI* restriction sites. This construct (pcDNA3-hP2X4) was used as template to amplify a DNA fragment encoding the hP2X4 extracellular domain (ECD;W50-I339) using the following primers: 5’-CACGCTAGCCATCACCATCACCATCACTGGGAAAAGGGCTACCAGGAAAC-3’ (forward),

and 5’-CACGGATCCTCAGATCATAGTGGGGATG-3’(reverse). This fragment was then subcloned into the prokaryotic expression vector pET11c (Novagen), in fusion with a N-terminal 6xHis tag. This vector (called pET-hisECD) was used for transformation of *E.coli BL21(DE3)*, and protein expression was induced. After purification with Ni-NTA agarose (Qiagen) under denaturing conditions, the HIS-ECD peptide was used for immunization of mice to develop mAbs. C57BL/6 mice (2 mice, 15-week-old females) were immunized three times with 40 µg purified hisECD protein intraperitoneally (i.p.) on days 0, 21 and 42. Complete Freund’s adjuvant (FA) was used for the first immunization and incomplete FA for the followings. On day 49 two mice with highest titre of antibodies against P2X4 were given a perfusion boost without adjuvant (50 µg recombinant ECD in PBS) and then the splenocytes were fused 4 days later on day 53. Spleen and bone marrow cells were removed from immunized mice and harvested by trituration under sterile conditions. The cells were fused with a HAT-sensitive mouse myeloma cell line Sp2/0 by using polyethylene glycol (PEG) method (Galfrè, Howe, Milstein, Butcher, & Howard, 1977; Milstein & Galfrè, 1981). Splenocytes from mice were mixed with the myeloma cells at ratios of 5:1. 7×10^5 myeloma cells were mixed with the bone marrow cells from 2 femurs. The suspension of fused splenocytes from one mouse was plated into ten 96-well tissue culture plates and the suspension of fused bone marrow cells into one microtitre plate (100 µl/well). Freshly prepared thymocytes (2×10^6 cells/ml) of a female SD rat were used as feeder cells in hybridoma cultures. Hypoxanthine-aminopterin-thymidine (HAT) medium (100 µl/well) was added after 24 hours. Hybridomas producing mAbs against hP2X4 ECD were identified with ELISA, and cloned by the limiting dilution method (Goding, J. W. 1980, 7007515). After a second screening based on ELISA and FACS, hybridomas were cultured in 10% FCS DMEM medium. The isotype of mAbs was determined with the Mouse Immunoglobulin Isotyping ELISA Kit (BD Biosciences). IgGs were purified from the supernatant of clones 19 (IgG2b) and 27 (IgG2b) using Pierce Protein G Agarose (Thermofisher Scientific), and conjugated with fluorescein isothiocyanate (FITC, Thermo Fisher Scientific)).

ELISA

ELISA was performed on 96-well plates, which were coated with 2.5 ug/ml HIS-ECD protein at 4°C overnight, washed with PBS/Tween 0.05% (PBS/Tw), and then blocked with 2% skim milk (Arla, Sweden) by overnight incubation. After washing, antibodies (mouse polyclonal

serum and hybridoma supernatants) were applied as a primary antibodies and incubated at + 4°C overnight followed by three washes. Polyclonal goat anti-mouse Ig-HRP diluted 1:3000

were added as secondary antibodies. The plates were incubated for one hour, washed and developed using 3,3´,5,5´-tetramethylbenzidine substrate in 0,1 M K-citrate buffer (pH 4.25). Optical densities (OD values) were detected by reading the plates on an ELISA plate reader (Thermo Scientific) at a wavelength of 450 nm.

Immunoprecipitation and Western blotting

For immunoprecipitation experiments shown in Figure 1 panel A, 80% confluent HEK cells ($4x10^5$ cells/well) cultured in 6-well plates were transfected with pcDNA3-hP2X4 DNA using polyethylenimine transfection reagent (Sigma-Aldrich). Cells were lysed 24 hours post transfection in ProteoYET Mammalian Cell Lysis Reagent (Thermo Fisher Scientific; 100 µl buffer per 106 cells) with 1/100 vol/vol Halt Protease Inhibitor cocktail (Thermo Fisher Scientific). Lysates were clarified by centrifugation at 12000xg and incubated for 2 hrs with primary antibodies e.g. 50 µl of hybridoma supernatant was added to 50 µl of cell lysate. Immunocomplexes were recovered by overnight incubation at 4°C with Protein G Agarose (50 µl of 50% bead slurry, Thermo Fisher Scientific), then the centrifuged pellet was washed 3 times with PBS and resuspended in 40 µl SDS-denaturing buffer. For Western blotting, the total protein lysates of P2X4-transfected cells or immunoprecipitation complexes resuspended in SDS-loading buffer (16 µl per lane) were separated on SDS-polyacrylamide (12%) gels and transferred onto a PVDF membrane (Millipore). Membranes were incubated with mouse polyclonal serum, or our mAbs, or rabbit anti P2X4 antibodies (Alomone), thereafter washed with PBS/Tw and incubated with HRP-conjugated goat anti- mouse IgG (Bio-Rad eBioscience), or goat anti rabbit IgG (Thermo Fisher Scientific), respectively, as secondary antibodies. Blots were developed by SuperSignal WestPico Chemiluminescent (Thermo Scientific).

For the experiments shown in Figure 1 panel B, $2x10^7$ cells from the three different cell lines were lysed in 1 ml lysis buffer containing Tris-HCl 20 mM, pH 7.4, 0.15M NaCl, 1% NP40 (IGEPAL® CA-630 NP 40), 1 mg/ml BSA and protease inhibitors (Roche). Cells were incubated 30 minutes in ice. Lysates were centrifuged at 600 g for 15 minutes at 4°C to remove nuclei and cellular debris. Supernatants were centrifuged at 12000 x g for 1 hour. Equal amounts of cell lysates were incubated overnight at 4°C with 20µl of protein-G agarose beads presensitized as indicated in the legend of figure 1. Protein G beads were washed three times and resuspended in loading buffer containing β-mercaptoethanol. Twenty millions HEK293 cells expressing the human or mouse P2X4 receptor mCherry were lysed in 1 ml lysis buffer as described above and equal amounts of cellular lysates were incubated overnight at 4°C with protein-G magnetic beads presensitized with mAb 27 or mAb 29. Protein G magnetic beads were washed three times and resuspended in loading buffer containing β- mercaptoethanol. Proteins from immunoprecipitates were then separated by SDS-PAGE and transferred to nitrocellulose membranes (Trans-Blot® TurboTM Mini Nitrocellulose Transfer Packs -Biorad) blocked with 5% BSA in Tris buffered saline containing 0.2% Tween 20 for 1 hour at 37°C. Blots were immunostained with primary antibodies at 4°C overnight and probed with secondary antibodies conjugated to horseradish peroxidase. Specific bands were visualised by enhanced chemiluminescence (Biorad) and read on ImageQuantTM LAS 500 (GE Healthcare life Sciences).

Inhibition of mAb27 binding to the P2X4receptor by unlabeled anti-hP2X4 mAbs

The following cell lines, HEK293 human P2X7R and HEK293 human P2X4-mcherry were treated with trypsin-EDTA for 10 minutes at 37°C. Cells were collected, washed in DMEM medium and spun down at 4°C. Cells were fixed and permeabilized one hour in ice as recommended by the manufacturer (eBiosciences). 1.5 X 10^6 cells were aliquoted in tubes and incubated with 0.6 micrograms of mAb27 FITC or 0.6 micrograms of isotype FITC control overnight at 4°C. For mAb inhibition of mAb27 binding, cells were preincubated with 2 micrograms of unlabelled mAb27 or mAb29 for at least 15 minutes in ice. Then, 0.6 micrograms of mAb27 FITC or 0.6 micrograms of isotype FITC control overnight at 4°C, were added in presence of unlabelled mAb inhibitor. Next morning, cells were washed twice in permeabilization buffer and analysed by flow cytometry after gating on mcherry positive cells.

RNA preparation and RT-QPCR

Total RNA was isolated from sorted cell pellets obtained after removal of the supernatant using RNeasy Mini kit (Qiagen, Helsinki, Finland) according to the instructions. ComplementaryDNA was synthesized with RevertAid TM reverse transcriptase (Thermo Fisher Scientific) according to the manufacturer's protocol. The PCR was performed using the following primers and 35 amplification cycles PCR amplification containing Taq polymerase (Thermo Fisher Scientific) was performed by 35 cycles consisting of 94 C for 30 seconds, 56 C for 30 seconds,72 C for 40 seconds

GAPDH: (F: 5' – CTG ACT TCA ACA GCG ACA CC – 3'; R: 5' – CTG ACT TCA ACA GCG ACA CC – 3'; product= 109 pb)
SIGLEC-8: (F: 5'-TGTTGACAGCACAGACCAGG-3'; R: -TCGATGTGTCCTACCCTCCT-3' -product= 138 pb)

Human tissue section staining

Cryosections (5 µm in diameter) were made from gall bladder surgical specimen that was isolated during routine surgery from patient with cholecystitis diagnosis. MilestonePrestoChillwas used for quick freezing of tissue sample. Sections were cut with Leica CM1950 and placed to cancer diagnostics Autofrost slide. Sections were washed 3x with TBS. Thereafter, sections were stained with anti-hP2X4 mAb27–FITC (1:1600; 200µl) and Siglec-8-PE (1:10; 50 µl, BioLegend) and washed again with 3x with TBS. Sections were stained for 10 min with Hoechst 33342 nucleic acid stain (1:100, 100 µl, Invitrogen), for cell- permeant nuclear counterstaining that emits blue fluorescence when bound to dsDNA. Sections were washed again 3x with TBS, and mounted with Dako fluorescence mounting medium.
