# Supplementary figures and images for: Human Peripheral Blood Eosinophils Express High Levels of the Purinergic Receptor P2X4
Source: Front Immunol. 2019 Sep 6;10:2074. doi: 10.3389/fimmu.2019.02074 (PMC6746186; doi:10.3389/fimmu.2019.02074)

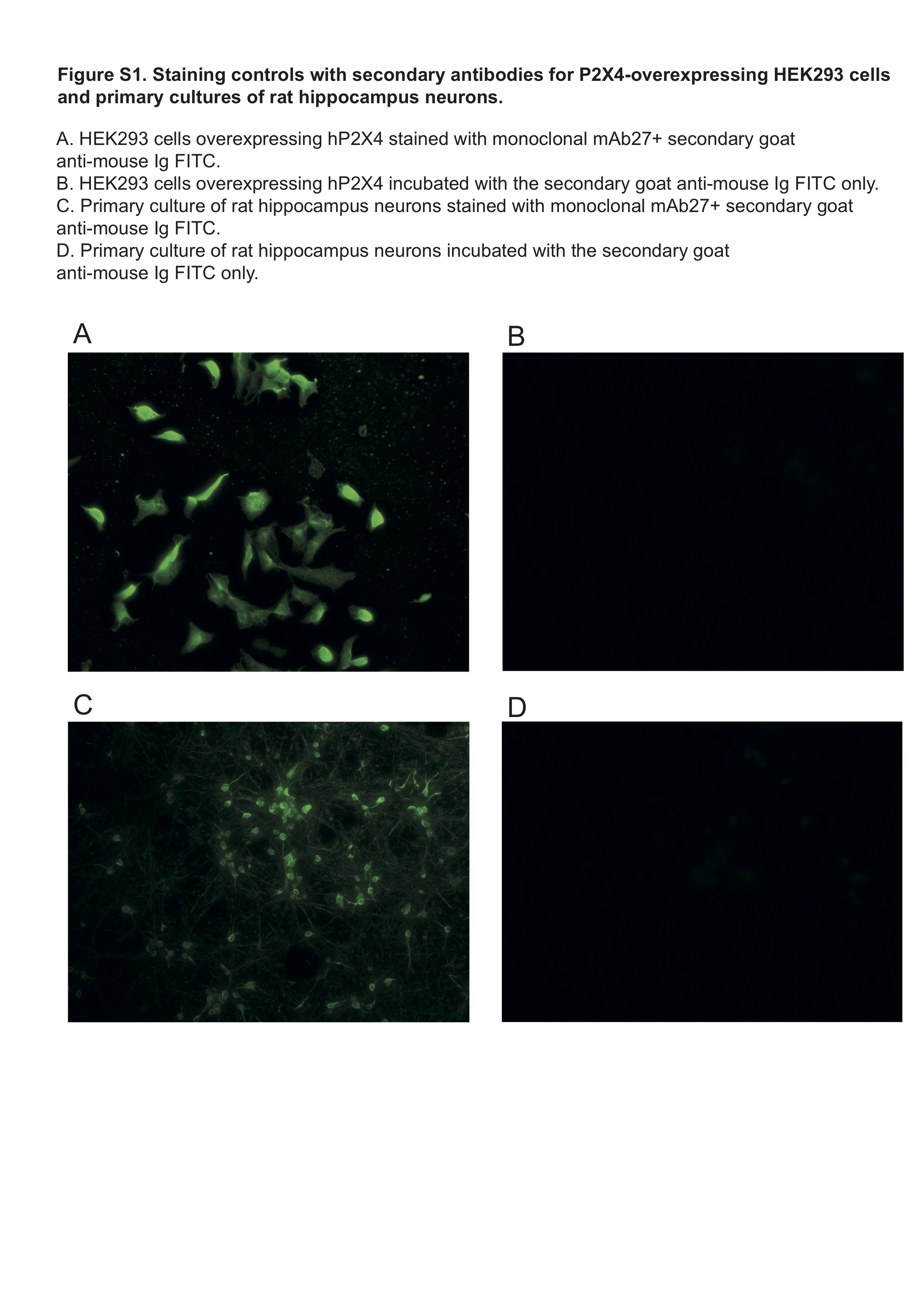

Supplement: Supplementary file 2 [file Image_1.TIFF]

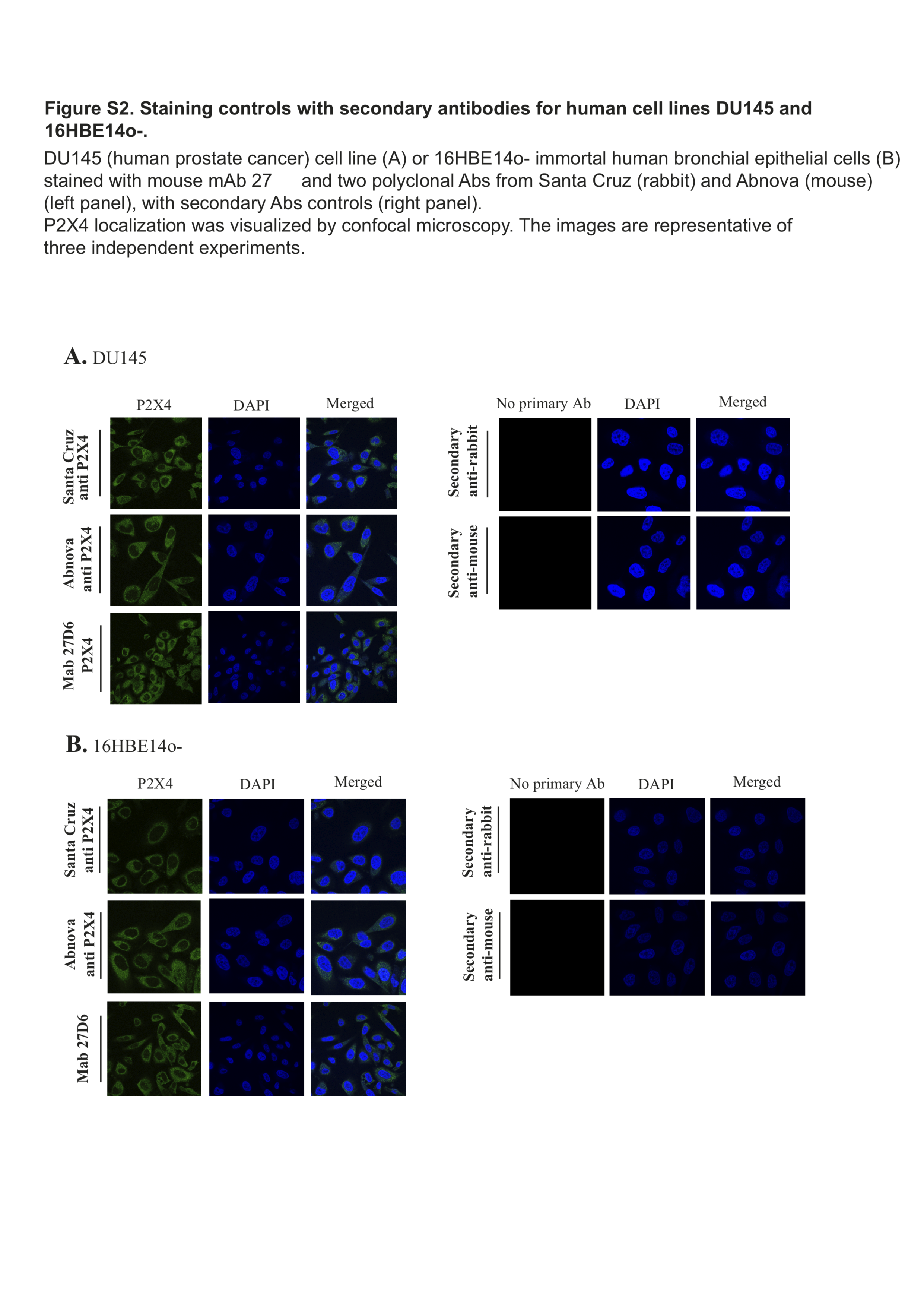

Supplement: Supplementary file 3 [file Image_2.tiff]

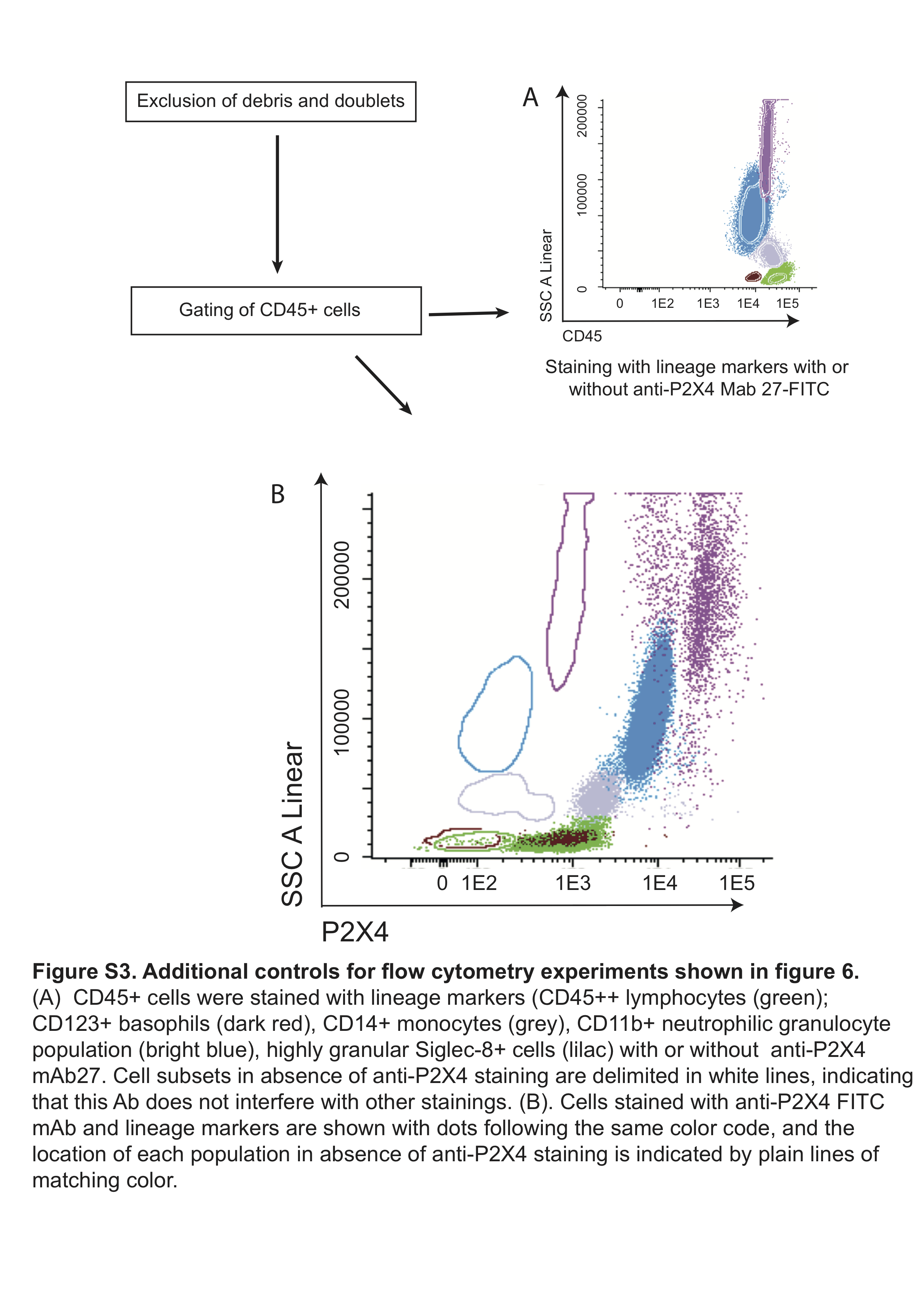

Supplement: Supplementary file 4 [file Image_3.TIFF]

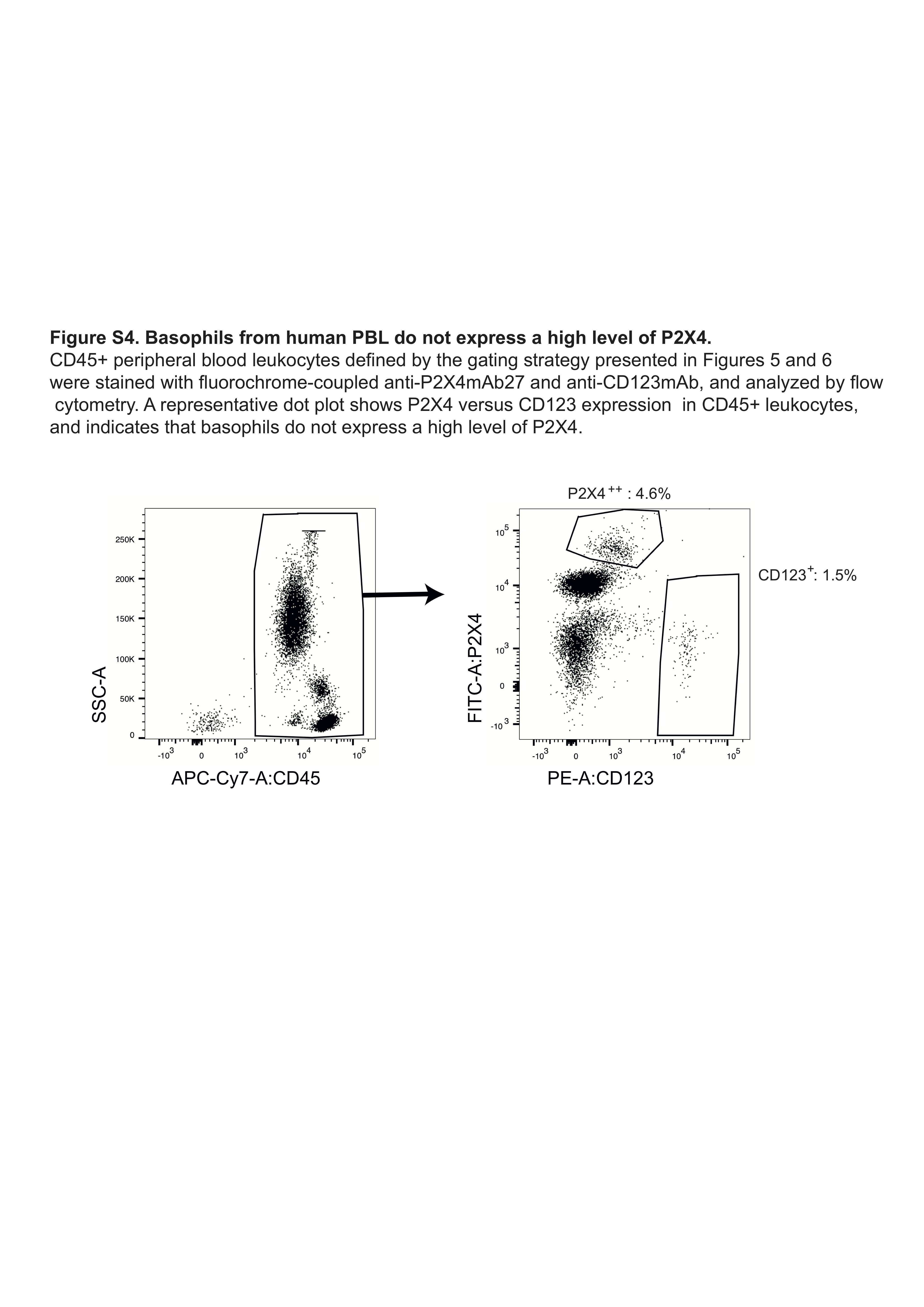

Supplement: Supplementary file 5 [file Image_4.TIFF]

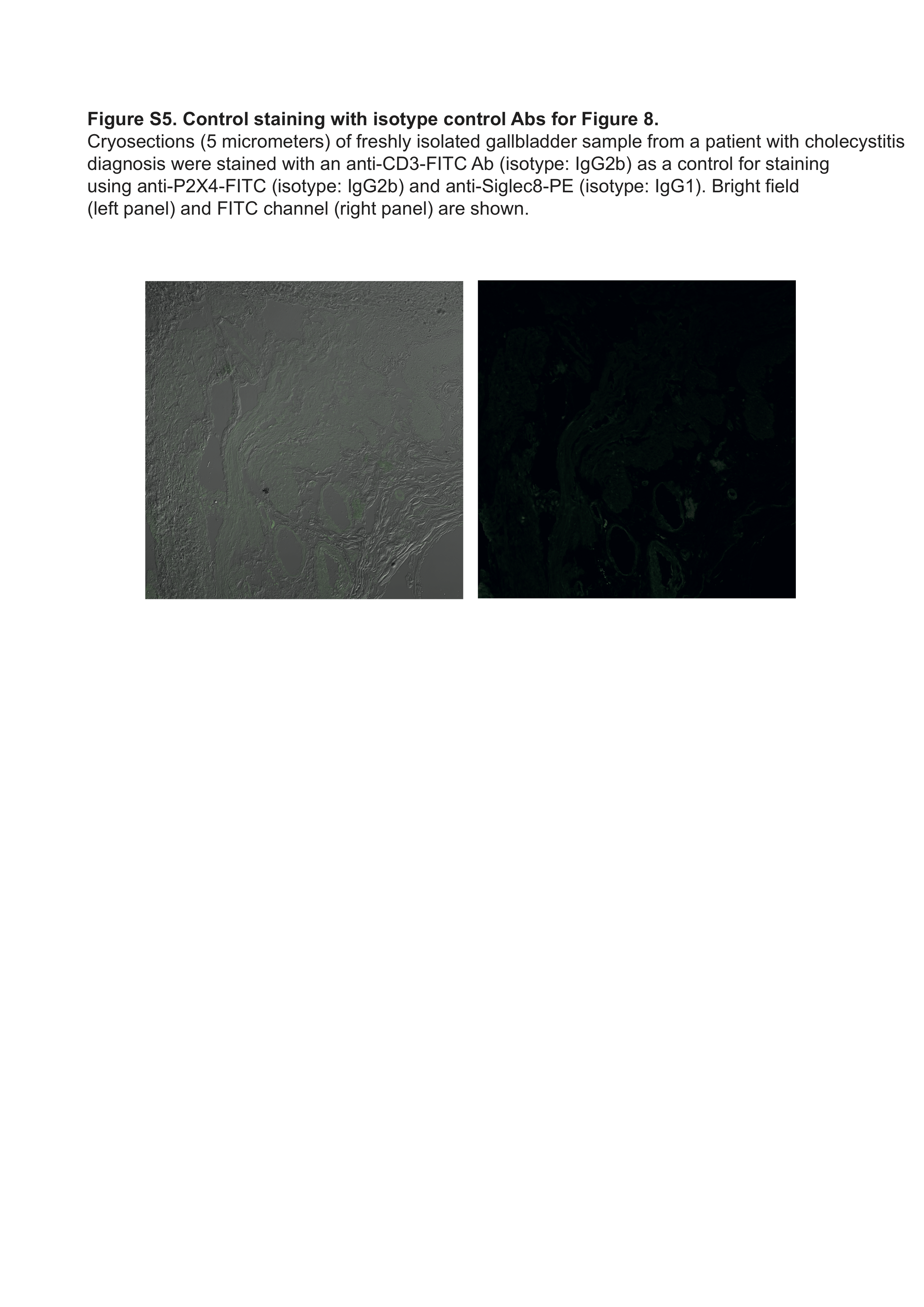

Supplement: Supplementary file 6 [file Image_5.TIFF]

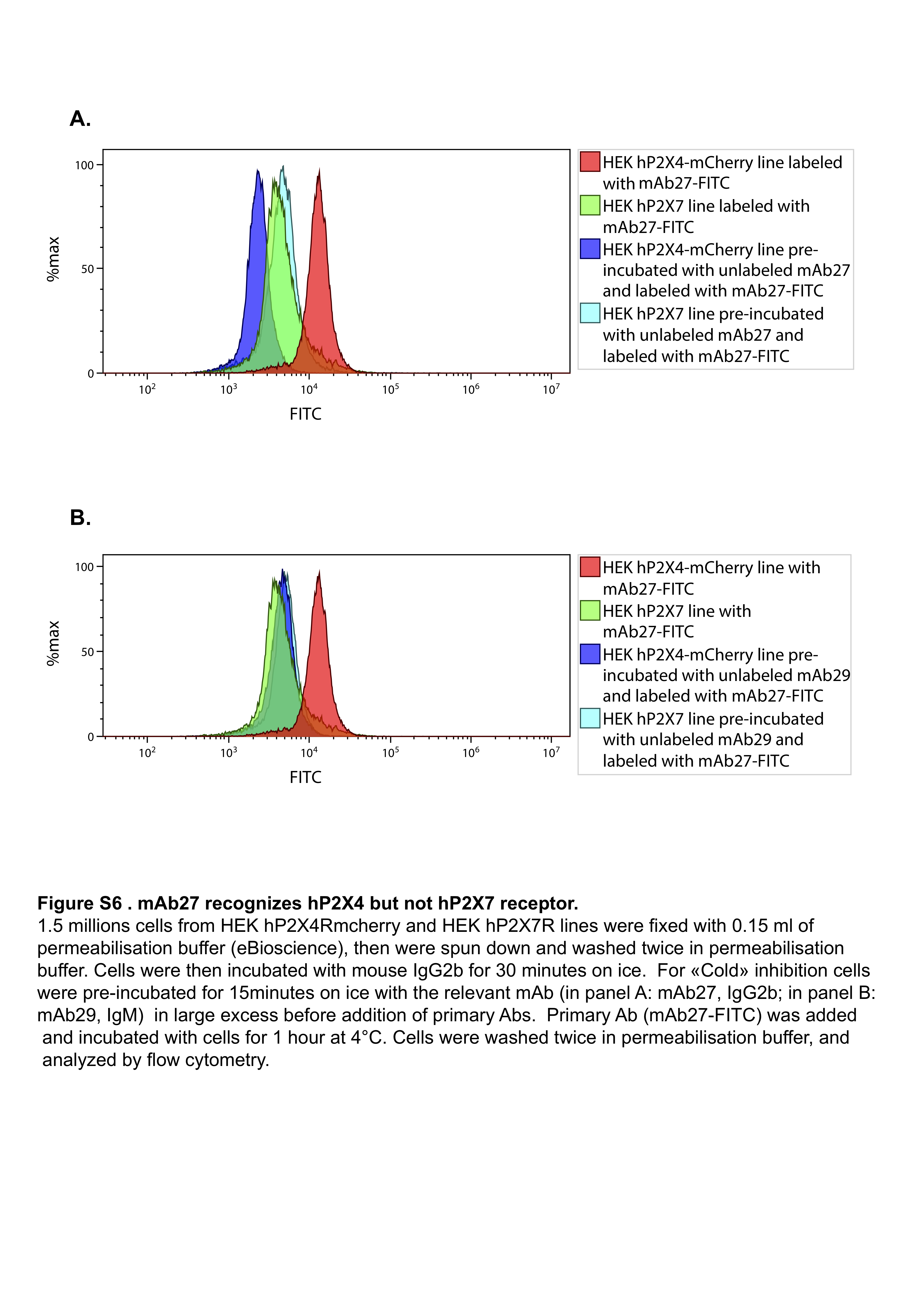

Supplement: Supplementary file 7 [file Image_6.TIFF]

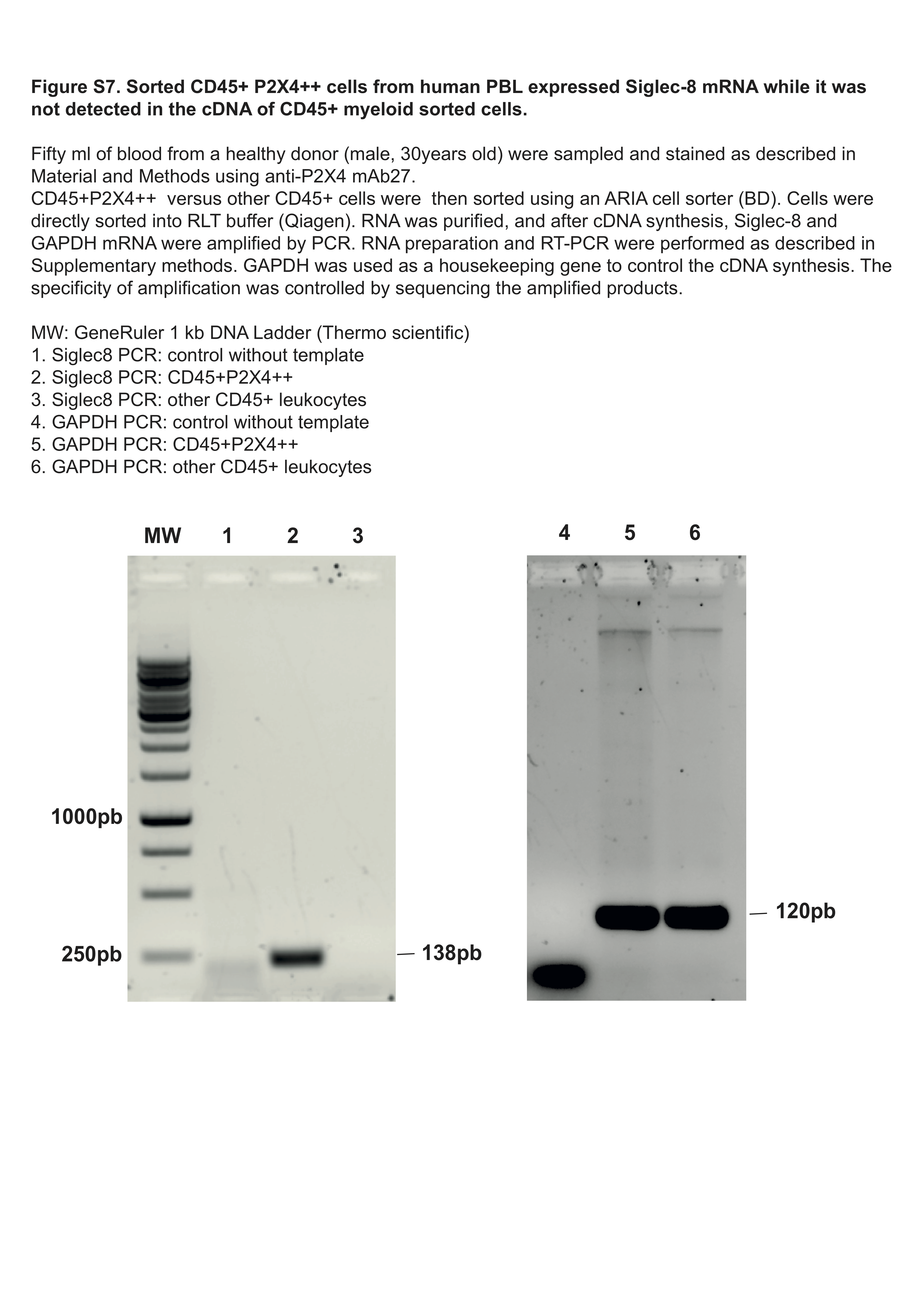

Supplement: Supplementary file 8 [file Image_7.TIFF]
